# Neuroimmunogastroenterology: At the Interface of Neuroimmunology and Gastroenterology

**DOI:** 10.3389/fneur.2020.00787

**Published:** 2020-07-31

**Authors:** John Michael S. Sanchez, J. Scott McNally, Melissa M. Cortez, James Hemp, Laura A. Pace, Stacey L. Clardy

**Affiliations:** ^1^Division of Microbiology and Immunology, Department of Pathology, University of Utah, Salt Lake City, UT, United States; ^2^Department of Radiology, Utah Center for Advanced Imaging Research, University of Utah, Salt Lake City, UT, United States; ^3^Department of Neurology, Imaging and Neurosciences Center, University of Utah, Salt Lake City, UT, United States; ^4^Division of Gastroenterology, Department of Internal Medicine, University of Utah, Salt Lake City, UT, United States; ^5^George E. Whalen Veterans Affairs Medical Center, Salt Lake City, UT, United States

**Keywords:** autonomic disease, neuroimmunology, gastroenterology, autoimmune disease, motility disorders

## Abstract

The central nervous system (CNS) is an important regulator of the gastrointestinal tract, and CNS dysfunction can result in significant and disabling gastrointestinal symptom manifestation. For patients with neuroimmunologic and neuroinflammatory conditions, the recognition of gastrointestinal symptoms is under-appreciated, yet the gastrointestinal manifestations have a dramatic impact on quality of life. The current treatment strategies, often employed independently by the neurologist and gastroenterologist, raise the question of whether such patients are being treated optimally when siloed in one specialty. Neuroimmunogastroenterology lies at the borderlands of medical specialties, and there are few resources to guide neurologists in this area. Here, we provide an overview highlighting the potential mechanisms of crosstalk between immune-mediated neurological disorders and gastrointestinal dysfunction.

## Introduction

The central nervous system (CNS) is an important regulator of the gastrointestinal tract, and CNS dysfunction can result in gastrointestinal symptom manifestation (1). Examples can be found in lesions of the hindbrain, such as in the medullary raphe nuclei that controls gastric motility and secretion through secretion of 5HT (5-hydroxytryptophan) and substance P (2, 3); midbrain, as seen in cerebellum-associated nausea and emesis related to motion sickness (4); and forebrain, including amygdala control of gastric secretion (5). While such conditions may be familiar to the practicing neurologist, neuroinflammatory-associated gastrointestinal disorders are less recognized.

Patients with inflammatory and autoimmune neurological disorders often have accompanying gastrointestinal symptoms, although the etiology of these gastrointestinal symptoms can vary. For example, a majority of multiple sclerosis patients report symptoms suggestive of a gastrointestinal motility disorder (6), likely related to involvement of the autonomic nervous system, while intractable nausea and vomiting in neuromyelitis optica spectrum disorder is directly attributable to central nervous system (CNS) lesions of the area postrema (7). For patients with other systemic neuroimmunologic and neuroinflammatory conditions, the recognition of gastrointestinal symptoms is likely under-appreciated, perhaps owing to more dramatic symptoms in other organ systems—yet the gastrointestinal manifestations of these disease processes can have a dramatic impact on quality of life.

Likewise, for patients presenting primarily with gastrointestinal symptoms, there is often an underappreciation of the neurologic, neuroimmune, and inflammatory nature of the condition. For example, transcriptomic analysis of patients with idiopathic gastroparesis reveal a clear role of the immune system in the disease process (8), and similarly, in patients with esophageal dysmotility disorders, there is a high rate of autoantibody detection (9, 10). Yet, most patients are treated surgically (11), rather than with immune-modulating therapy. The current treatment strategies, often employed independently by the neurologist and gastroenterologist, raise the question of whether such patients are being treated optimally when siloed in one specialty—especially if there is not a recognition of the potential neurologic, neuroimmune, immunologic and inflammatory underpinnings of the symptoms. Indeed, neuroimmunogastroenterology lies at the borderlands of medical specialties, and there are few currently available resources to guide neurologists and gastroenterologists in this area. Here, we provide an overview of recent literature highlighting the potential mechanisms of crosstalk between immune-mediated neurological disorders and gastrointestinal dysfunction. In addition, we report three clinical cases to emphasize the diagnostic and therapeutic avenues to evaluate and treat gastrointestinal complaints in patients with inflammatory and autoimmune neurological disorders.

## Literature Review

### General Mechanisms of Neuroimmune-Gastrointestinal Crosstalk

**Figure 1:**
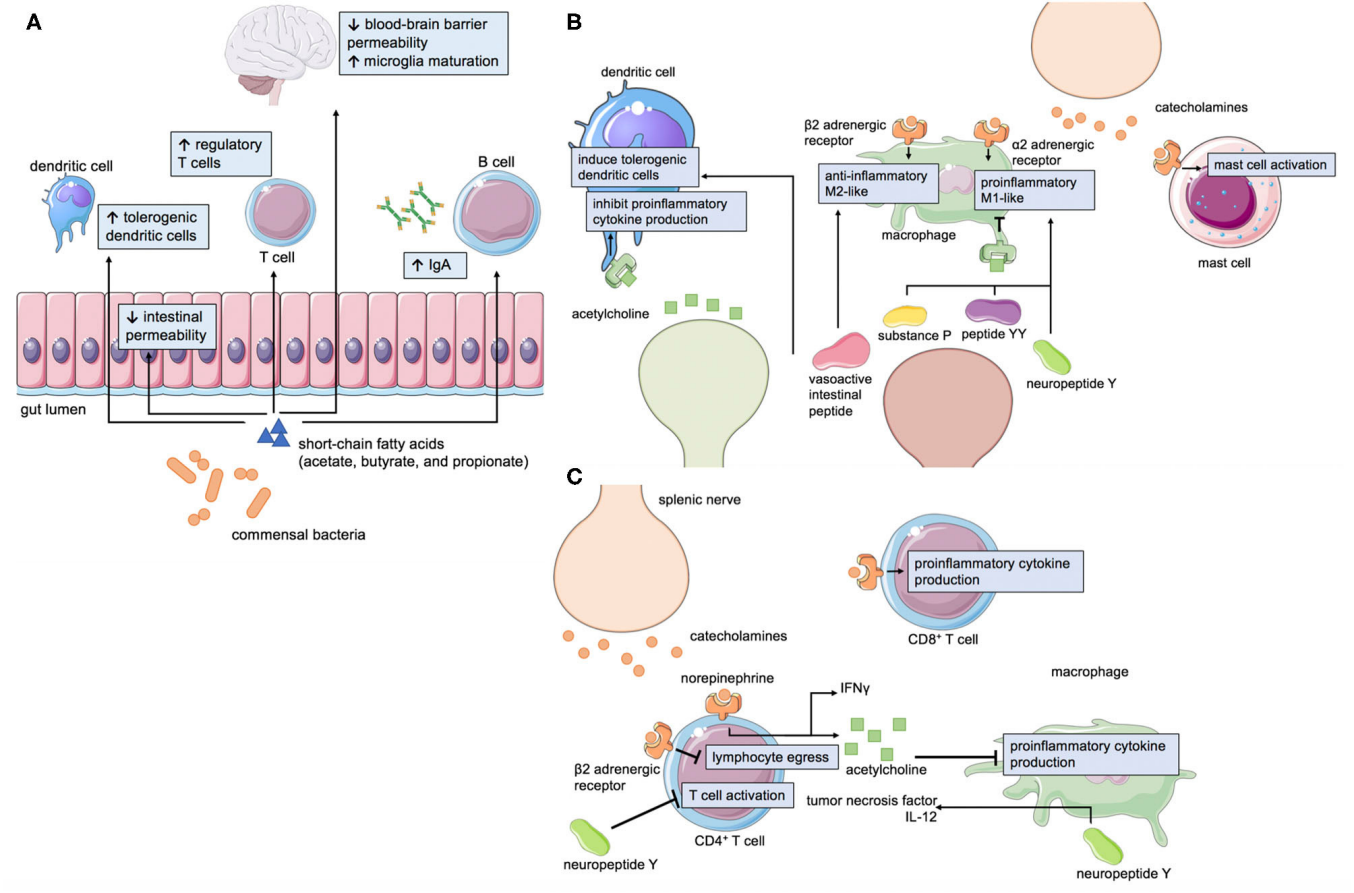
Mechanisms of neuroimmune-gastrointestinal crosstalk. **(A)** Short-chain fatty acids (SCFAs) such as acetate, butyrate, and propionate are metabolites generated by commensal gut bacteria that affect intestinal permeability, immunity, and CNS physiology. Neurotransmitters and neuropeptides can influence the function of **(B)** innate and **(C)** adaptive immune cells.

The brain-gut axis incorporates bi-directional signals from the central nervous system, autonomic nervous system, enteric nervous system, gut microbiota, and immune system to respond to the dynamic needs of human physiology. The sympathetic and parasympathetic divisions of the autonomic nervous system provide efferent cues to modulate gastrointestinal motility, secretion, and blood flow to the gastrointestinal tract (12). In contrast, afferent neurons of the enteric nervous system relay information to the CNS regarding the enteric milieu, including mechanical distension and the fed/fast state of the gastrointestinal tract (13). Within the gastrointestinal tract itself resides the densest collection of commensal microorganisms, the gut microbiota, which produce neuroactive molecules and have substantial influence on mucosal immunity (Figure 1A) (14). In fact, the majority of the human body's immune cells are found within gut-associated lymphoid tissue and are key mediators of the brain-gut axis.

Cells of the innate immune system are classically understood to mount the initial immune response to pathogens, however the crosstalk between cells of the immune and nervous systems are functionally significant in the gastrointestinal tract. Catecholamines, a major class of sympathetic neurotransmitters, have been shown to differentially induce pro- (M1-like) and anti- (M2-like) inflammatory macrophages in the intestine (15). The effect of catecholamine signaling appears to be context-dependent as activation of α2 adrenoreceptors promotes the production of proinflammatory cytokines (16, 17) whereas activation of β2 adrenoreceptors promotes the production of anti-inflammatory cytokines (18). Mast cells express adrenergic receptors, and signaling through these receptors can modulate the secretion of mast cell products (19). In contrast, the major parasympathetic neurotransmitter, acetylcholine, has been shown to decrease pro-inflammatory cytokine production in macrophages and dendritic cells, among other immune cell types (18, 20, 21). Serotonin, 95% of which is synthesized in the gut (22), modulates the polarization of macrophages (23) and phagocytosis (24). Mast cells themselves have the ability to produce serotonin (25), which can directly affect gastrointestinal motility, and histamine, which can activate sensory neurons in the gastrointestinal tract (26). Neuropeptides, including substance P, neuropeptide Y and peptide YY, can enhance the production of pro-inflammatory cytokines in monocytes (27) and enhance phagocytosis in macrophages (28). Vasoactive intestinal peptide (VIP), another neuropeptide, induces regulatory dendritic cells (29, 30) which can then go on to induce regulatory T cells. VIP can also reduce TGF-β1 (31), which can drive pro-inflammatory T-helper-17 cells, and increase IL-10 production in macrophages (32). Thus, neurons of the enteric nervous system and intestine-resident innate immune cells are intimately associated through bidirectional signaling (Figure 1B).

The adaptive immune system is also involved in neuro-immune crosstalk in the gastrointestinal tract (Figure 1C). Interestingly, some T cells are intrinsically capable of producing acetylcholine via choline acetyltransferase (21). Acetylcholine-producing T cells can be activated by efferent signals from the vagus nerve to the splenic nerve (21). Conversely, afferent vagal signaling can result in anti-inflammatory effects (33). Catecholamine signaling via β2-adrenergic receptors interact with chemokine receptors to control T and B cell migration (34) and can differentially modulate cytokine production in different T cell subsets (35, 36). Similarly, dopamine can modulate T cell migration and cytokine secretion (37). Neuropeptides can also modulate the production of cytokines by T cells (38, 39). VIP is one such example that increases inflammatory cytokine production in T cells (40). Overall, the large collection of innate and adaptive immune cells that reside in the gut are linked to cues from the nervous system and the microbiota to maintain homeostasis in a constantly changing environment.

### The Role of the Gut Microbiota and Gut Permeability in Neuroinflammatory Disease

From birth, and likely even *in utero*, the community of bacteria, archaea, viruses, and fungi that colonize the human gut play an important role in the physiology of the gastrointestinal, immune, and nervous systems (41). Commensal microorganisms and their metabolites can influence gastric secretion and motility by directing serotonin production; modulate the differentiation and function of immune cells; and increase neurogenesis (42). The epithelium of the gastrointestinal tract is critical to maintaining a barrier between the luminal contents of the intestines and the rest of the body, however this barrier has selective permeability that allows for the absorption of nutrients as well as certain microbial products (43, 44). In some neuroinflammatory conditions, the permeability of the intestinal barrier is increased that can allow for pathogenic translocation of gut microorganisms and microbiota-derived products into circulation that promote inflammation (45). Conversely, certain commensal microorganisms including certain strains of *Lactobacillus* and *Bifidobacterium* have the ability to upregulate the expression of tight junction proteins between epithelial cells and strengthen the gut barrier (46). Evidence for the role of the gut microbiota and the intestinal barrier in neuroinflammation has been found in a number of disease states including autism spectrum disorder, multiple sclerosis, and Parkinson's disease. Understanding the mechanisms by which the gut microbiota and intestinal permeability influences neuroinflammation is an intense area of research.

Autism spectrum disorder is a continuum of neurodevelopmental conditions characterized by repetitive behaviors, altered sociability, and language difficulties (47). Gastrointestinal dysfunction and increased permeability of the gastrointestinal barrier is a common comorbidity of autism (48). Patients with autism spectrum disorder also display alterations in gut bacterial communities, including a lower abundance of *Akkermansia, Bacteroides, Escherichia coli*, and *Enterococcus* and a higher abundance of *Faecalibacterium* and *Lactobacillus*, compared to typically developing controls (49). Gnotobiotic mice that receive fecal microbiota from autism spectrum disorder patients exhibit exacerbated autism-like behaviors compared to mice that receive fecal microbiota from typically developing controls (50). Specific bacteria, such as *Bacteroides fragilis* and *Lactobacillus reuteri*, have been shown to modulate mouse models of autism spectrum disorder by reducing gut inflammation, permeability and targeting oxytocinergic and dopaminergic networks (51–53). Importantly, microbiota-based therapies including fecal microbiota transplant, antibiotics, and probiotics have shown benefit in small clinical studies (54–56).

Multiple sclerosis is an immune-mediated demyelinating disease of the CNS and the most common cause of non-traumatic physical disability in young adults (57). Gastrointestinal dysmotility and increased permeability is prevalent in patients with multiple sclerosis and in experimental autoimmune encephalomyelitis (EAE), the most commonly used mouse model of multiple sclerosis (58–61). A number of studies have reported changes in specific strains of bacteria such as elevated *Methanobrevibacter* and *Porphyromonas* and decreased *Bacteroides* and *Lactobacillus* in multiple sclerosis patients compared to healthy matched controls (62–67). Additionally, disease-modifying therapies for multiple sclerosis have been shown to alter gut microbial composition, though the functional consequences of these drug-induced changes are not known (65, 68). Gnotobiotic mice colonized with multiple sclerosis-associated fecal microbiota exhibit more severe EAE compared to gnotobiotic mice colonized with fecal microbiota from matched controls, suggesting that the gut microbiota can influence multiple sclerosis (66, 67). Indeed, specific bacteria including *Bacteroides, Lactobacillus*, and *Prevotella* have been shown to protect against EAE by modulation of the immune response, including induction of regulatory T cells and suppression of proinflammatory T-helper (Th) type 1 and Th17 cells (69–73). Importantly, pilot studies of probiotic administration or dietary invention with associated downstream effects on the gut microbiota have been shown to benefit multiple sclerosis patients (74–76).

Parkinson's disease is a neurodegenerative disease associated with pathological aggregation of α-synuclein in the CNS (77). Degeneration of the dopaminergic neurons of the substantia nigra pars compacta leads to the characteristic symptoms of Parkinson's including tremor, muscle rigidity, and impaired balance (77). Interestingly, patients with Parkinson's disease exhibit gastrointestinal inflammation and increased intestinal permeability years prior to neurological deficits and α-synuclein can be found early in the enteric nervous system and the glossopharyngeal and vagal nerves (78, 79). Indeed, patients with Parkinson's disease exhibit alterations in certain gut commensal taxa including decreased Prevotellaceae and increased Verrucomicrobiaceae (80–83). Utilizing a murine model of synucleinopathy, colonization of gnotobiotic mice with the fecal microbiota of Parkinson's disease patients accelerated the accumulation of α-synuclein in the CNS compared to mice colonized with microbiota from matched controls (84). Short-chain fatty acids, which are derived from the gut microbiota, was also shown to be sufficient to accelerate accumulation of α-synuclein in the CNS, though the mechanism is unclear (84). Interestingly, the human commensal bacterium, *Enterococcus faecalis*, has the ability to metabolize levodopa, a dopaminergic agent used to treat Parkinson's disease, and decrease its bioavailability in an animal model (85). Together, these data suggest a functional role of the gut microbiota in Parkinson's disease pathogenesis and treatment.

The above examples demonstrate the wide-reaching influence the gut microbiota has on neurological disorders of varying etiologies. The ability of the gut microbiota to modulate the immune system, produce neuroactive compounds, affect gut barrier function, among other functions, adds a layer of complexity to neuroinflammatory diseases. The mechanisms of communication between the gut microbiota and the CNS along the gut-brain axis remain to be fully elucidated. Still, the positive results from pilot trials using probiotics, antibiotics, and fecal microbiota transfer in certain neurological conditions suggests that microbiota-based therapies may be on the horizon for specific contexts.

### Neurological Manifestations of Gastrointestinal Disease

In the opposite direction, certain immune-mediated gastrointestinal diseases, including inflammatory bowel disease (IBD) and celiac disease, have been shown to have neurological involvement. While the exact mechanisms linking gastrointestinal inflammation to neurological dysfunction remain to be elucidated, future investigation will be critical in addressing these manifestations.

IBD, which includes ulcerative colitis and Crohn's disease, is characterized by chronic inflammation of the gastrointestinal tract. Imaging studies have demonstrated increased white matter lesions (86–88), decreased gray matter volume (88, 89), and increased axonal damage (88) in patients with IBD compared to healthy matched controls. While other studies have failed to identify these IBD-associated neurological changes (90, 91), this may be due to differences in the severity of IBD at the time of imaging. Indeed, gray matter atrophy has been reported to correlate with IBD duration (89). The etiology of these structural brain lesions is not completely understood, though several mechanisms have been proposed. These include thromboembolism, vasculitis, neurotoxic inflammation, chronic pain-induced excito-toxicity, demyelination, iatrogenic progressive multifocal leukoencephalopathy, and opportunistic infections (92, 93). IBD-associated brain lesions can be asymptomatic, however the association of IBD with peripheral neuropathy, myopathy, myelopathy, and cerebrovascular disease suggest that IBD can lead to true dysfunction of the nervous system (94).

Unlike IBD, where the cause of gastrointestinal inflammation is often not clear, patients with celiac disease exhibit gastrointestinal inflammatory disease triggered by dietary gluten (95). Celiac disease is frequently associated with neurological symptoms ranging from ataxia, neuropathy, headache, cognitive impairment, and psychiatric disorders, though celiac disease is not likely the primary cause of these conditions (96). Neurological involvement has been attributed to nutritional deficiencies, including deficiencies in vitamin B12, vitamin E, folate, zinc, or copper (96), due to due to loss of brush border proteins and enzymes needed for the absorption of these nutrients, and also inflammation of the small intestine (97). Other deficiencies are also common but not often directly related to neurologic symptoms, including reduced levels of iron, folate, vitamin D, and magnesium (97). Overlapping nervous system autoimmunity may also contribute to neurological symptoms as anti-transglutaminase antibodies, a biomarker of celiac disease, have been reported in the CNS of celiac disease patients (98) Indeed, intraventricular injection of anti-transglutaminase antibodies into mice results in ataxia (99). Auto-antibodies to gangliosides have also been reported (100); the significance remains unclear but the generation of anti-ganglioside antibodies is speculated to be linked to the intestinal immune response to ingested gluten. Similar to ganglioside generation in acute immune-mediated neuropathies, where molecular mimicry between gangliosides and bacterial or viral oligosaccharides is proposed as a mechanism (101) in celiac disease as well (100). Following a gluten-free diet, which alleviates gastrointestinal symptoms of celiac disease, has mixed success in relieving the neurological manifestations of the disease (102–105). This observation reinforces the possibility that celiac disease may be an association of some neurologic symptoms but not the primary cause. For example, in some hereditary ataxia syndromes, CD antibodies are more commonly found than the normal population (106) Whether primary, secondary, or otherwise genetically associated, recognition and treatment of underlying celiac disease is worthy of investigation and treatment.

In animal models, gastrointestinal inflammation is often accompanied by systemic inflammation, chronic pain and stress, and nutritional deficiencies that can impede neurological function (107). While unethical to model chronic stress in humans, adequate intervention to disrupt systemic inflammation (and chronic stress as able) by early detection and mechanistic investigation of neurological dysfunction in the context of immune-mediated gastrointestinal diseases will be key.

### Functional Gastrointestinal Disorders

Among the most common gastroenterology diagnoses are functional gastrointestinal disorders (108). According to the most recent ROME IV criteria on functional gastrointestinal disorders, these are classified by symptoms related to any combination of gastrointestinal motility disturbance, visceral hypersensitivity, altered mucosal and immune function, altered gut microbiome, or altered CNS processing (109). While it is acknowledged that functional gastrointestinal disorders may be associated with processes such as motility disturbances or altered immune function, the recommendation by the ROME committee is to conduct a careful physical examination and cost-efficient investigation, with the diagnosis of a functional disorder relying largely on symptom description and duration, rather than objective findings. The ROME committee also reminds the clinician to acknowledge the worsening of certain symptoms with stress, without themselves acknowledging that the fundamental role of the autonomic nervous system in responding to both physical and emotional stress. Treatment recommendations most commonly include education, reassurance, and symptom management. This approach leaves the vast majority of patients undiagnosed and improperly treated, neglecting potential mechanism-directed therapeutic options. In contrast, it is our experience that a true etiology can be identified in many individuals with a diagnosis of a functional gastrointestinal disorder, if an expanded investigation is undertaken.

Irritable bowel syndrome (IBS) serves as the prototypical functional gastrointestinal disorder, as it is the most prevalent of the functional gastrointestinal disorders. IBS is estimated to affect ~11% of the world's population and is defined by abdominal pain and a change in bowel function (110). The diagnostic criteria for IBS include recurrent abdominal pain, on average at least 1 day per week in the last 3 months, associated with two of the following three conditions: (1) pain with defecation, (2) change in frequency of stool, or (3) change in the appearance of stool. An astute clinician can quickly realize that this simply categorizes individuals based on symptoms, and does not provide insight into the pathophysiologic determinants of the symptoms. Despite the recognition that genetic, environmental, and immune factors can increase the risk of functional gastrointestinal disorders, very few patients will undergo a thorough clinical evaluation. Within this paradigm, patients may be diagnosed with functional gastrointestinal disorders, with the true diagnosis never identified, leading to sub-optimal care. It is within this clinical space that the Gastroenterologist and Neurologist working together can provide true diagnoses and improve clinical care.

The inflammatory component of IBS has been recognized since early histological observations of increased inflammatory cells, including mast cells and T cells, in the intestinal mucosa of IBS patients (111, 112). These leukocytes are located in close proximity to nerves of the enteric nervous system that can respond to immune products such as cytokines and chemokines (113, 114). The role of inflammation is also substantiated by genome-wide association studies of IBS patients, identifying major risk alleles in immune-related genes such as TNFSF15 (115), which encodes for the cytokine tumor necrosis factor-like ligand 1A, and TLR9 (116), which encodes for a Toll-like receptor that senses microbe-derived DNA. IBS has also been linked to autoimmune conditions, including rheumatoid arthritis and psoriasis, though neural autoantibodies are only rarely found in IBS patients (117, 118). Still, stress commonly exacerbates IBS symptoms, which may be due to catecholamine-mediated induction of inflammation (119). Thus, the immune system emerges as a critical effector in IBS that can integrate signals from the nervous system and the gut microbial community to give rise to disease.

### Gastroparesis

Similar to IBS, gastroparesis is defined as a syndrome of delayed emptying of solid gastric contents without mechanical obstruction. The prevalence of gastroparesis is estimated to be 9.6 per 100,000 for men and 37.8 per 100,000 for women (120). Patients with gastroparesis exhibit symptoms including nausea, early satiety, postprandial fullness, gastroesophageal reflux, abdominal pain, and often vomiting. While diabetes and surgical trauma have been identified as major etiologies for gastroparesis, idiopathic gastroparesis is by far the most common (121). Gastroparesis is reported in 20% of individuals with a ROME IV diagnosis of functional dyspepsia (122), highlighting the difficultly in distinguishing gastroparesis from functional gastrointestinal disorders. Within idiopathic gastroparesis, immunologic, neurologic, neuroimmunologic, neuromuscular, connective tissue, genetic, and metabolic dysfunction may underlie the gastroparesis, further highlighting the missed opportunities for identifying the true diagnosis. Thus, by an error of attribution, a diagnosis of a functional gastrointestinal disorder limits our ability to appropriately investigate and treat these patients.

Among the underappreciated mechanisms of gastroparesis, neuroimmunological etiologies such as infectious and autoimmune should be considered. Interestingly, some patients develop gastroparesis after acute viral infections, including Epstein-Barr, Herpes simplex, and Varicella Zoster virus (123). Viral triggering of neuroimmune pathology (124) and/or autoimmunity (125) is a well-studied phenomenon that can lead to gastroparesis due to immune-mediated damage of the enteric nervous system. In addition, gastroparesis is associated with a number of autoimmune conditions including autoimmune gastritis, Sjogren's syndrome, and scleroderma (126–128). Indeed, inflammatory cytokines and reactive species, including interleukin-6, interleukin-1β, tumor necrosis factor-α, and inducible nitric oxide synthase, have been associated with impaired intestinal motility (129). Although most cases of gastroparesis are categorized as idiopathic, neuroimmunological etiologies explain at least some of these cases, and could provide opportunity for therapeutic intervention.

### Autoimmune Gastrointestinal Dysmotility

Autoimmune gastrointestinal dysmotility (AGID) is an autoimmune dysautonomia that remains underappreciated within the realm of gastroenterology. AGID presents with findings of abnormal gastrointestinal motility along with symptoms of nausea, vomiting, abdominal pain, constipation, and diarrhea. Greater than 70% of these individuals will have serologic evidence of detectable autoantibodies and others may have evidence of myenteric inflammation on full thickness biopsies from the gastrointestinal tract (130). The incidence and prevalence of AGID is unknown, as it is not routinely screened for in clinical practice, however the disease appears to be more prevalent in females (131). Patients with AGID may exhibit autonomic nervous system abnormalities limited to the gastrointestinal tract or systemic dysautonomia (130, 131). Some AGID patients also have overlapping autoimmune conditions such as Graves' disease, Celiac disease, lupus, and type I diabetes (130, 131). AGID can be idiopathic, meaning that the cause of autoantibody elevations is not yet understood, however, some cases may have a paraneoplastic etiology related to an underlying malignant process. Increased detection of AGID—and subsequent immune-directed treatment—has the potential to significantly improve outcomes and quality of life for patients with an otherwise refractory gastrointestinal motility disorder.

### Diagnostic Considerations

Scintigraphy based tests are considered the gold-standard method of measuring gastrointestinal transit (132), however these tests are limited in that they can only provide information related to transit. Furthermore, only scintigraphy-based gastric emptying studies are available at most centers, with small intestinal and colonic scintigraphy limited to only very specialized centers. This translates to underdiagnosis of the vast majority of small intestinal and colonic dysmotility. Manometric based testing and wireless motility capsule (WMC) analysis can both provide information regarding intraluminal pressure measurements and coordination of muscular contractions. Until very recently, antroduodenal-jejunal and colonic manometry were considered the gold-standard for diagnosing complicated gastrointestinal motility disorders, as it was thought this technology could differentiate between a myopathic and neuropathic pathology. However, recent systematic comparison of full thickness small intestinal histopathology to antroduodenal-jejunal manometry could not reliably differentiate myopathic and neuropathic gastrointestinal disorders of motility (133). Therefore, in some individuals the identification of a gastrointestinal motility disorder, whether made by manometry-based studies or WMC, should prompt the consideration for full thickness biopsy of the gastrointestinal tract to aid in diagnosis. The WMC provides important measures of regional and global gastrointestinal transit times, intraluminal pressure measurements, pH, and temperature measurements (134, 135) and has been incredibly useful in helping to diagnose many cases of AGID and characterizing the gastrointestinal motility disturbances secondary to underling neurologic disorders. An additional advantage of the WMC, over small intestinal and colonic scintigraphy, is that it can be performed outside of highly specialized centers.

Serology is a useful tool to identify autoimmune processes. While there is a vast array of neuronal-specific autoantibodies (136), certain autoantibodies are especially useful in the context of neuroimmune-mediated gastrointestinal disease (Table 1). Classically, these anti-neuronal antibodies are thought of as paraneoplastic processes that arise against onconeural antigens expressed by the tumor environment (170), however these autoantibodies can arise even in the absence of a tumor (171). Antibodies associated with AGID include: type 1 anti-neuronal nuclear autoantibody, ganglionic nicotinic acetylcholine receptor antibody, voltage-gated potassium channel-complex antibody, N-type calcium channel antibody, P/Q-type calcium channel antibody, muscle acetylcholine receptor antibody, striational antibody, collapsing response-mediator protein-5 antibody, and glutamic acid decarboxylase 65-kilodalton isoform antibody (131). Other autoantibodies associated with gastrointestinal dysmotility include: peripherin antibody, Purkinje cell cytoplasmic antibody type 1, and smooth muscle L-type calcium channel antibody. Antibodies that target cell surface epitopes, such as receptors and channels, commonly play a direct role in the pathogenesis of neurological dysfunction (172). For example, transfer of purified ganglionic nicotinic acetylcholine receptor antibody to rodents can recapitulate dysautonomia (144). Conversely, antibodies that target intracellular epitopes are largely thought to be a marker of disease generated from the release of autoantigens during neurodegeneration (172). Serology is limited to probing the humoral arm of the immune response and does not necessarily predict response to immunomodulation. Still, serology for neuronal antibodies may have utility in the setting of significant gastrointestinal symptoms unresponsive to conventional therapy.

**Table 1:** Neural autoantibodies associated with gastrointestinal dysmotility.

**Antibody**	**Antigen function**	**Cancer association**	**Phenotype**	**Mechanism**	**Responsive to Immunomodulation**	**References**
**AGID-associated**						
Collapsin response-mediator protein-5 (CRMP5) antibody	Axon guidance and neurite outgrowth signaling	Lung cancer, thymoma	Dementia, ataxia, myelopathy, chorea, seizures, neuropathic pain and gastroparesis	Axonal injury due to cytotoxic immune cells; no evidence for direct role in pathogenesis	Yes	(137)
Contactin-associated protein 2 (CASPR2) antibody	Scaffolding protein associated with voltage-gated potassium channels	Not common	Limbic encephalitis, seizures, cognitive deficits, cerebellar dysfunction, peripheral nerve hyperexcitability, autonomic dysfunction, and neuropathic pain	Proposed to alter CASPR2-mediated cell-cell interactions; evidence for direct role in pathogenesis	Yes	(138–143)
Ganglionic nicotinic acetylcholine receptor antibody	Ligand-gated ion channel that responds to acetylcholine	Adenocarcinoma	Severe widespread dysautonomia, including impaired pupillary light reflex, anhidrosis, and intestinal dysmotility	Impairs transmission of autonomic synapses; significant evidence for direct role in pathogenesis	Yes	(144–146)
Glutamic acid decarboxylase 65-kilodalton isoform (GAD65) antibody	Forms gamma aminobutyric acid (GABA)	Not common	Limbic encephalitis, epilepsy, cerebellar ataxia, and stiff-person syndrome	Proposed to inhibit GAD65 enzymatic activity and GABA synthesis; limited evidence for a direct role in pathogenesis	Yes	(147–150)
Leucine-rich glioma-inactivated 1 (LGI1) antibody	Secreted glycoprotein that regulates voltage-gated potassium channels and a-amino-3-hydroxy-5-methyl-4-isoxazolepropionic acid (AMPA) receptors	Not common	Limbic encephalitis, seizures, cognitive disturbance, psychiatric symptoms	Proposed to interrupt LGI1-receptor interactions, alter voltage-gated potassium channels, and impair AMPA receptor function; evidence for direct role in pathogenesis	Yes	(136, 141–143, 151, 152)
Muscle acetylcholine receptor antibody	Mediate acethylcholine response at the neuromuscular junction	Thymoma	Myasthenia gravis	Destruction of muscle acetylcholine receptors by crosslinking leading to internalization and degradation, complement-mediated lysis of the postsynaptic membrane, and direct inhibition of acetylcholine receptor; significant evidence for direct role in pathogenesis	Yes	(153)
N-type calcium channel antibody	Regulates calcium influx in response to action potential	Small-cell lung cancer; less common compared to P/Q-type calcium channel antibody	Gastrointestinal tract dysmotility, Lambert-Eaton myasthenic syndrome	Not clear	Yes	(154–156)
P/Q-type calcium channel antibody	Regulates calcium influx in response to action potential	Small-cell lung cancer	Paraneoplastic cerebellar degeneration, Lambert-Eaton myasthenic syndrome including proximal muscle weakness, reduced tendon reflexes, and autonomic dysfunction	Blocks calcium influx leading to reduced acetylcholine release from the presynaptic membrane; antibody-induced neuronal death; evidence for a direct role in pathogenesis	Yes	(157–159)
Striational antibody	Targets contractile skeletal muscle components including titin, myosin, actin, a-actinin, and ryanodine receptor	Thymoma	Myasthenia gravis	Presence associated with more severe myasthenia gravis, but no evidence that antibodies have a direct role in pathogenesis	Yes	(160, 161)
Type 1 anti-neuronal nuclear autoantibody (ANNA-1/anti-Hu)	Family of RNA-binding proteins	Small-cell lung cancer	Autoimmune encephalomyelitis, cerebellar degeneration, motor neuron disease, and gastrointestinal dysmotility	Associated with neuronal degeneration; limited evidence for direct role in pathogenesis	Usually no	(162–165)
**Other**						
Peripherin antibody	Neuronal intermediate filament protein	Not common	Dysautonomia (particularly gastrointestinal dysmotility) and endocrinopathy	Autoreactive cytotoxic T cells specific to peripherin-derived peptides; no evidence for direct role in pathogenesis	Unknown	(166)
Purkinje cell cytoplasmic antibody type 1 (PCA-1/anti-Yo)	Targets cerebellar degeneration-related protein, a transcriptional regulator	Gynecologic and breast cancers	Paraneoplastic cerebellar degeneration	Proposed to disrupt calcium homeostasis and lead to cell death; limited evidence for direct role in pathogenesis	Yes, limited benefit	(167)
Smooth muscle L-type calcium channel antibody	Regulates calcium influx in response to action potential	Not common	Gastrointestinal dysmotility	Activates smooth muscle L-type calcium channels, some evidence for direct role in pathogenesis	Unknown in humans, yes in preclinical models	(168, 169)

Various imaging modalities can be used to capture signs of inflammation and gastrointestinal dysfunction. Abdominal CT is also commonly used to visualize the bowels and adjacent structures to uncover obstructions and other anatomical problems affecting the gastrointestinal tract (173). Similarly, FDG-PET/CT is an excellent method for the non-invasive quantification of bowel inflammation, and has been studied extensively in patients with IBD (174). These imaging modalities are critical to localize the inflammatory insult, however imaging must be combined with other diagnostics to determine the underlying cause. Specific to inflammation of the nervous system, inflammation can be more challenging to detect unless profound, but MRI of the brain and spinal cord (with contrast) can be a useful test to detect neuroinflammatory lesions on T2-weighted and fluid attenuated inversion recovery (FLAIR) sequences (175). Likewise, FDG-PET/CT can be useful, and specific to autoimmune encephalitis, FDG-PET/CT has been found to be more often abnormal than initial EEG, MRI, and CSF studies in neurology inpatients with autoimmune encephalitis, with brain region hypometabolism the most frequently observed pattern (176). Obtaining FDG-PET/CT scanning, either of the brain or body, can be quite challenging, as it is often not covered by insurance for investigation of inflammatory or immune-mediated conditions in the United States (177) or internationally.

Autonomic testing is a critical diagnostic to assess for dysautonomia. The sensitivity and quantitative nature of autonomic testing allows for the localization and severity of autonomic dysfunction to be measured. Broadly, autonomic testing evaluates adrenergic, cardiovagal, and sudomotor functions (178). Adrenergic function is assayed by monitoring blood pressure recovery during Valsalva, monitoring blood pressure and heart rate in response to head-up tilt, measuring plasma norepinephrine in response to standing, and measuring the uptake of labeled norepinephrine analogs (179). Cardiovagal function is assayed by monitoring heart rate in response to deep breathing and Valsalva (180). Sudomotor function is assayed by quantitative sudomotor axon reflex test and thermoregulatory sweat test (180). In addition to these standard autonomic tests, other tests including pupillometry, testing lacrimal and salivary gland production, and urodynamic studies can provide additional read outs of autonomic function (181–183). Combined, these tests can help delineate whether gastrointestinal dysmotility is derived from lesions of the autonomic nervous system and can also monitor patient response to treatment.

Proper workup of overlapping neurological and gastrointestinal symptoms requires the use of various diagnostic modalities (Table 2). Combined, the goals of these modalities are to localize the neurological insult, identify an immune etiology, and quantify gastrointestinal dysfunction.

**Table 2:** Diagnostic modalities useful in neuroimmunogastroenterological conditions.

**Diagnostic test**	**Indications**	**Result**	**Limitations**
Brain and Spine MRI	CNS inflammation	Localizes inflammation in the CNS	Limited information on cause of inflammation
Serology	Autoimmune process	Identifies autoantibodies	Quantifies only humoral immune response
Autonomic Testing	Multi-system dysautonomia	Localizes and quantifies dysautonomia	Limited information on cause of dysautonomia
PET-CT	Neoplastic process	Highlights metabolic activity and can identify neoplasm and inflammation	Expensive, radiation exposure, insurance coverage
Wireless motility capsule	Intestinal dysmotility	Measures intestinal transit times, intraluminal pressure	Potential for capsule retention in gastrointestinal tract, insurance coverage
Scintigraphy-based gastrointestinal studies	Intestinal dysmotility	Quantifies gastrointestinal motility	Small intestinal and colonic scintigraphy limited to specialized centers
Antroduodenal-jejunal and colonic manometry	Intestinal dysmotility	Quantifies intraluminal pressure and coordination of contractions	Patient discomfort
Abdominal CT	Gastrointestinal symptoms	Localizes gastrointestinal pathology	Radiation exposure

### Therapeutic Considerations

Mild cases of IBS are commonly managed with diet and lifestyle modifications by avoidance of trigger foods and management of stress, though more severe manifestations of IBS may require drugs such as prokinetics, anticholinergics, opioid antagonists, antidiarrheals, or antibiotics (184). Significantly, mast cell-targeted drugs have shown to be beneficial in IBS. Disodium cromoglycate is an example of such an agent, which reduces the release of the major mast cell effector tryptase in jejunal biopsies, and provided symptomatic benefit to patients with diarrhea-predominant IBS (185, 186). Ebastin, a histamine 1-receptor antagonist, targets the signaling of another major mast cell product, histamine, and was found to relieve abdominal pain associated with IBS (187).

While gastroparesis is treated symptomatically with antiemetic and prokinetic medications or with gastric electrical stimulation devices (188), a subset of gastroparesis patients fail to respond to these interventions. In a retrospective study of drug and device resistant gastroparesis patients with evidence of neuroinflammation, as determined by the presence of anti-glutamic acid decarboxylase antibodies and inflammation on full-thickness gastric biopsy, intravenous immunoglobulin improved nausea, vomiting, and abdominal pain (189). This was followed up by a prospective study in a small cohort of refractory gastroparesis patients with evidence of immune dysfunction and similarly found symptomatic benefit of IVIG (190).

Patients with AGID are often identified after failing symptomatic treatment of their gastrointestinal dysmotility. In the setting of autoimmune autonomic disorders, plasma exchange has been shown to be efficacious in relieving gastrointestinal dysmotility due to dysautonomia (191). IVIG and IV methylprednisolone have also been used as diagnostic trials in patients with suspected AGID with some success in addressing gastrointestinal symptoms (130). As an example of a targeted approach, an AGID patient positive for ganglionic neuronal acetylcholine receptor was treated with pyridostigmine, an acetylcholinesterase inhibitor, resulting in alleviated gastrointestinal symptoms (192). Due to the low prevalence of AGID, studies of AGID therapy have been limited to a non-randomized, retrospective design. Still, the promising reports of immunomodulation in this population should encourage future randomized, controlled trials.

With appropriate recognition and characterization, inflammatory and autoimmune neurological disorders afford the treating physician an opportunity to improve the quality of life of patients with concomitant neurologic and gastrointestinal dysfunction.

## Illustrative Case Series

We now present three clinical vignettes to illustrate our interdisciplinary approach to the diagnosis and treatment of neuroimmunogastrointestinal disorders.

### Case 1

**Figure 2:**
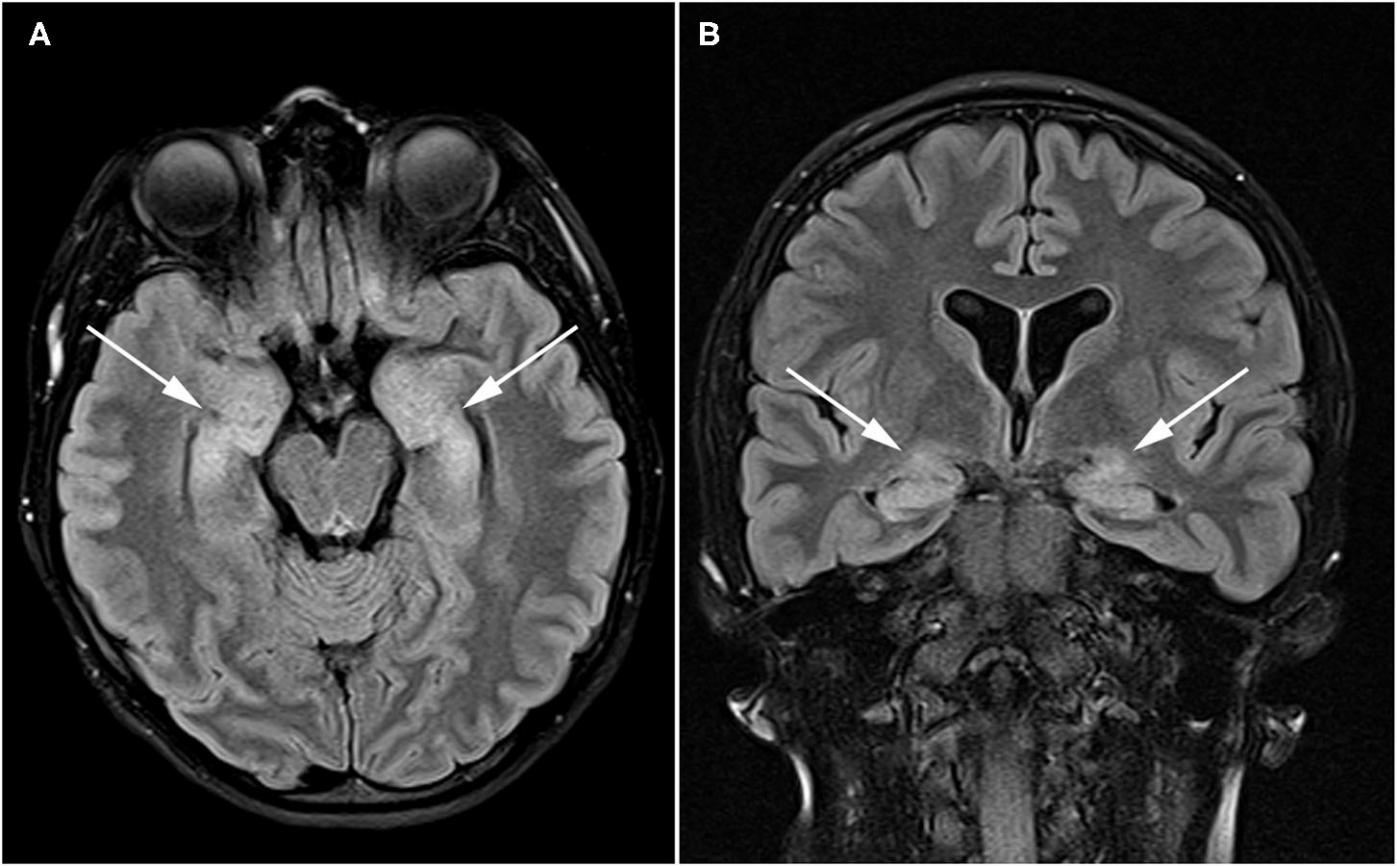
Patient with autoimmune enteric nervous system dysfunction and limbic encephalitis. Axial **(A)** and coronal **(B)** FLAIR images demonstrate high signal throughout the bilateral hippocampi and amygdala (arrows) compatible with limbic encephalitis.

A 21-year-old female with known systemic autoimmunity including autoimmune interstitial lung disease (long-standing positive cyclic citrullinated peptide (CCP) antibody and rheumatoid factor (RF); phenotypically meeting Sjögren's syndrome criteria) and a history of neurofibromatosis type I, presented to Neurogastroenterology Clinic with atypical, episodic spells of intractable nausea and vomiting accompanied by profound dizziness. A brain MRI with and without contrast was obtained during the course of evaluation, and revealed abnormal T2/FLAIR hyperintense signal within the bilateral hippocampi suggestive of autoimmune limbic encephalitis (Figure 2). Since childhood, she had reported heartburn symptoms, frequent nausea with vomiting, motion sickness, and chronic constipation. However, these episodic spells represented a dramatic change from her baseline symptoms. At the time of her initial evaluation in Neurogastroenterology Clinic, she already had been given a diagnosis of a functional nausea and vomiting disorder, and her interstitial lung disease had been stabilized with long-standing mycophenolate mofetil. Given the description of her episodes of profound nausea and vomiting, she was referred for an MRI of the brain (results discussed above) and EEG, which was normal, along with an evaluation of gastrointestinal motility. She completed SmartPill WMC (Medtronic, Dublin, Ireland) testing with small intestinal, colonic, and global gastrointestinal motility delays noted, with preservation of appropriate colonic responses to sleep and waking. She also underwent anorectal manometry, given her reports of significant straining and sensation of incomplete evacuation with defecation, which revealed high anal sphincter pressures (maximum resting pressure 157 vs. 40–100 mm Hg; maximum squeezing pressure 298 vs. 100–180 mm Hg), visceral hypersensitivity, and evidence of an evacuation disorder. She also underwent standardized autonomic reflex testing including 10- min 70° head-up tilt (HUT), heart rate variability with deep breathing (HRDB), Valsalva maneuver, quantitative pupilometry, and sudomotor testing (Q-SWEAT). While blood pressure and heart rate responses to HUT and pupillometry were normal, Q-SWEAT responses (right forearm sweat output 0 mL/cm^2^ vs. 0.08 mL/cm^2^, right proximal leg sweat output 0.09 mL/cm^2^ vs. 0.19 mL/cm^2^, right distal leg 0.1 mL/cm^2^ vs. 0.14 mL/cm^2^), HRDB (11.86 beats per minute (BPM) vs. >14 BPM) and Valsalva ratio (1.42 vs. > 1.46) were reduced, suggesting an underlying autonomic neuropathy. Given her clinical symptoms, abnormalities noted on brain imaging, and the finding of an underlying autonomic neuropathy, her immunotherapy was changed to rituximab, resulting in a dramatic improvement, but not complete resolution, of her gastrointestinal symptoms. Addition of linaclotide 290 mcg once daily improved her complaints of chronic constipation associated with straining and sensation of incomplete evacuation with defecation, which then nearly resolved the remainder of her gastrointestinal symptoms.

This case highlights the danger of categorizing patients exhibiting chronic gastrointestinal symptoms with a functional disorder, or similarly, misattributing a significant change in symptomology to evolution of pre-existing gastrointestinal symptom. It is important to consider the possibility of an underlying neurological disorder when faced with atypical gastrointestinal complaints (193). This case also confirms that insults to the temporal lobe can mimic true gastrointestinal symptoms (194–196). Utilization of manometry and autonomic testing allowed for the acquisition of objective data regarding autonomic and enteric nervous system function. Imaging was also instrumental in supporting a central nervous system inflammatory process, justifying a diagnostic trial of rituximab, which alleviated many of her gastrointestinal symptoms and severe episodes of profound nausea and vomiting with dizziness.

### Case 2

**Figure 3:**
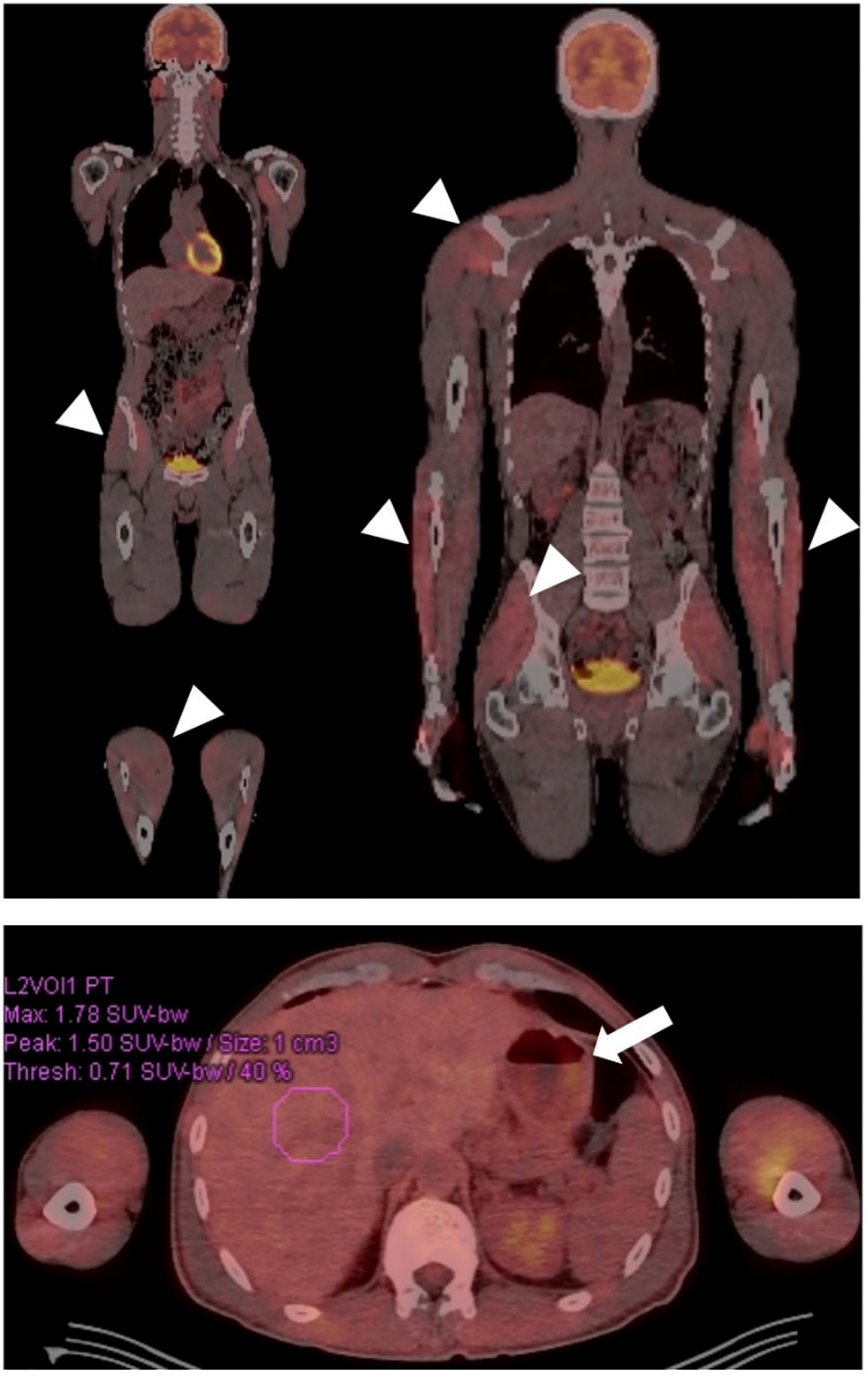
Patient with autoimmune gastroparesis and diffuse myositis. Body FDG-PET revealed diffuse uptake throughout the musculature compatible with the patient's known myositis (arrow heads). Gastroparesis was also incidentally demonstrated, given presence of a large amount of stomach contents despite fasting for >12 h (arrow).

**Figure 4:**
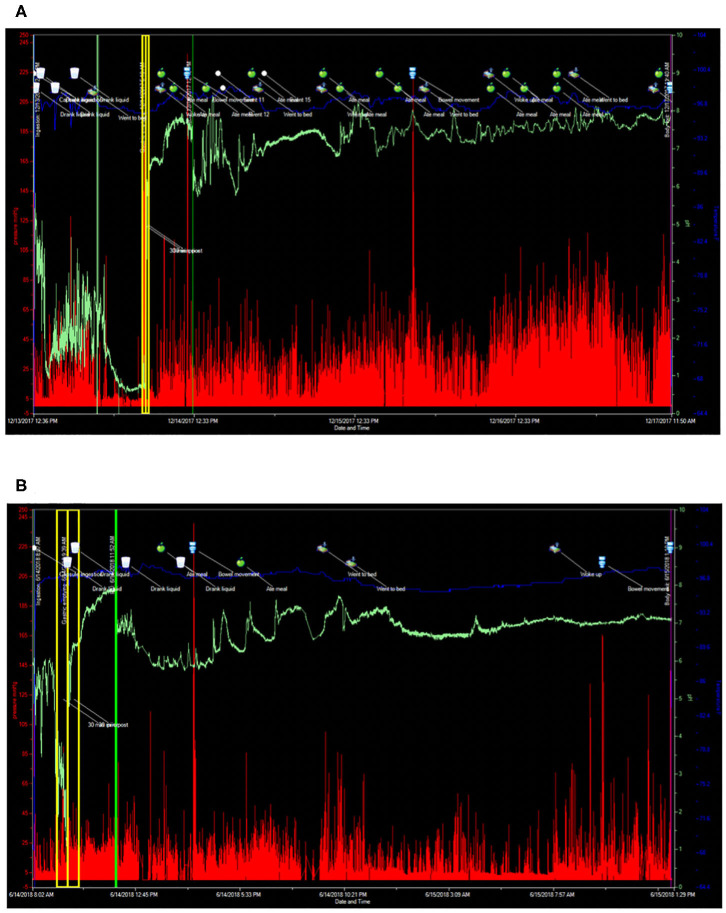
SmartPill wireless motility capsule studies before and after immunomodulatory therapy in a patient with autoimmune myositis. **(A)** Prior to commencement of Rituximab. Gastric emptying time 16 h, 38 min (normal <4 h), small intestinal transit time 7 h (normal <6 h), colonic transit time 71 h, 20 min (normal <59 h), and global gastrointestinal transit time 94 h, 58 min (normal <73 h). **(B)** SmartPill wireless motility capsule after commencement of steroids and Rituximab. Gastric emptying time 1 h, 31 min (normal <4 h), small intestinal transit time 2 h, 12 min (normal <6 h), colonic transit time 25 h, 27 min (normal <59 h), and global gastrointestinal transit time 29 h, 12 min (normal <73 h). For each SmartPill study below, the blue tracing represents temperature measurements, the green tracing is pH, the vertical red lines represent intraluminal pressure measurements with the height of the red line corresponding to the intensity of the pressure measurement. The vertical yellow lines correspond to the 30 min before and 30 min after gastric emptying. The vertical green line corresponds to passage of the capsule from the small intestine into the colon. The cup symbol marks fluid ingestion, the apple symbol marks solid food ingestion, the toilet symbol marks bowel movements, the bed symbol marks periods of sleep and waking.

A 43-year-old male with a remote history of Burkitt's lymphoma treated with R-CHOP (rituximab, cyclophosphamide, doxorubicin hydrochloride, vincristine sulfate, and prednisone), along with multiple courses of intrathecal methotrexate and history of small bowel resection, presented to Neuroimmunology Clinic with worsening fatigue and paresthesias over the course of 5 years. A lumbar puncture performed to rule out recurrence of CNS cancer cells revealed elevated protein levels (52 mg/dL vs. 14–45 mg/dL). Prior to presentation, he had experienced a 15-pound unintentional weight loss associated with complaints of early satiety, post-prandial fullness, abdominal bloating, constipation, and anorexia. Serological testing revealed positive titers of anti-nuclear antibodies (ANA) (1:160 vs. <1:40; speckled pattern) and borderline levels of myositis specific antibodies (anti-SAE1, SUMO activating enzyme, and anti-MDA5, CADM-140), however he did not clearly fall into a specific myositis profile, as he had a normal creatine kinase and electromyography (EMG). As part of his clinical evaluation, he underwent cross-sectional imaging of his abdomen that demonstrated dilation of the stomach suggestive of profound gastroparesis. Given his symptoms, and the concern for recurrence of malignancy, a whole-body PET-CT was obtained, which demonstrated diffuse, non-specific muscle activity, along with marked gastric distention and retention of gastric contents despite being NPO for greater than 12 h prior to the imaging study (Figure 3). This finding was again suggestive of severe gastroparesis. Therefore, a SmartPill WMC study was performed, and demonstrated prolonged gastrointestinal transit times in all regions (stomach >16 h, normal <5 h; small intestine 7 h, normal 2.5–6 h; and colon >71 h, normal <59 h) and globally (>94 h, normal <73 h) (Figure 4). Given concerns for an autoimmune/autoinflammatory myositis as contributing to symptoms, abnormalities noted on PET-CT, and gastrointestinal dysmotility, he was started on infusions of intravenous methylprednisolone (eventually transitioning to oral prednisone) and rituximab. This resulted in a dramatic improvement in fatigue and paresthesias, along with gastrointestinal symptoms including early satiety, post-prandial fullness, abdominal bloating, constipation, and anorexia. Follow up SmartPill analysis three months post-treatment was again abnormal, however there was demonstrated improvements in transit times which correlated with significant improvements in gastrointestinal symptoms (Gastric emptying time, 1 hour 31 minutes; small intestine transit time, 2 h 12 minutes; colonic transit time, 25 h, 27 minutes; and global gastrointestinal transit time, 29 h 12 minutes). He was slowly tapered off 7.5 mg prednisone daily over the course of 12 months, with continuation of rituximab monotherapy and ongoing improvement of symptoms.

This case emphasizes the benefits of coordinated efforts between a neuroimmunologist and a neurogastroenterologist, and the utility of PET-CT and the SmartPill wireless motility capsule, to provide objective data in an otherwise difficult to diagnose immune-mediated myositis. The history of Burkitt's lymphoma initially raised the concern for an underlying paraneoplastic process. PET-CT was helpful in ruling out recurrence of malignancy and in providing objective evidence of myositis without the need for muscle biopsy (Figure 3); an unanticipated finding was the evidence of gastroparesis, which guided further testing and led to confirmation of this previously unappreciated aspect of his disease burden. Using wireless motility capsule measurements, we demonstrated diffuse gastrointestinal dysmotility that dramatically improved after commencement of immune-modulating therapy. The objective improvements in gastrointestinal motility correlated with the dramatic improvement in the patient's gastrointestinal symptoms.

### Case 3

**Figure 5:**
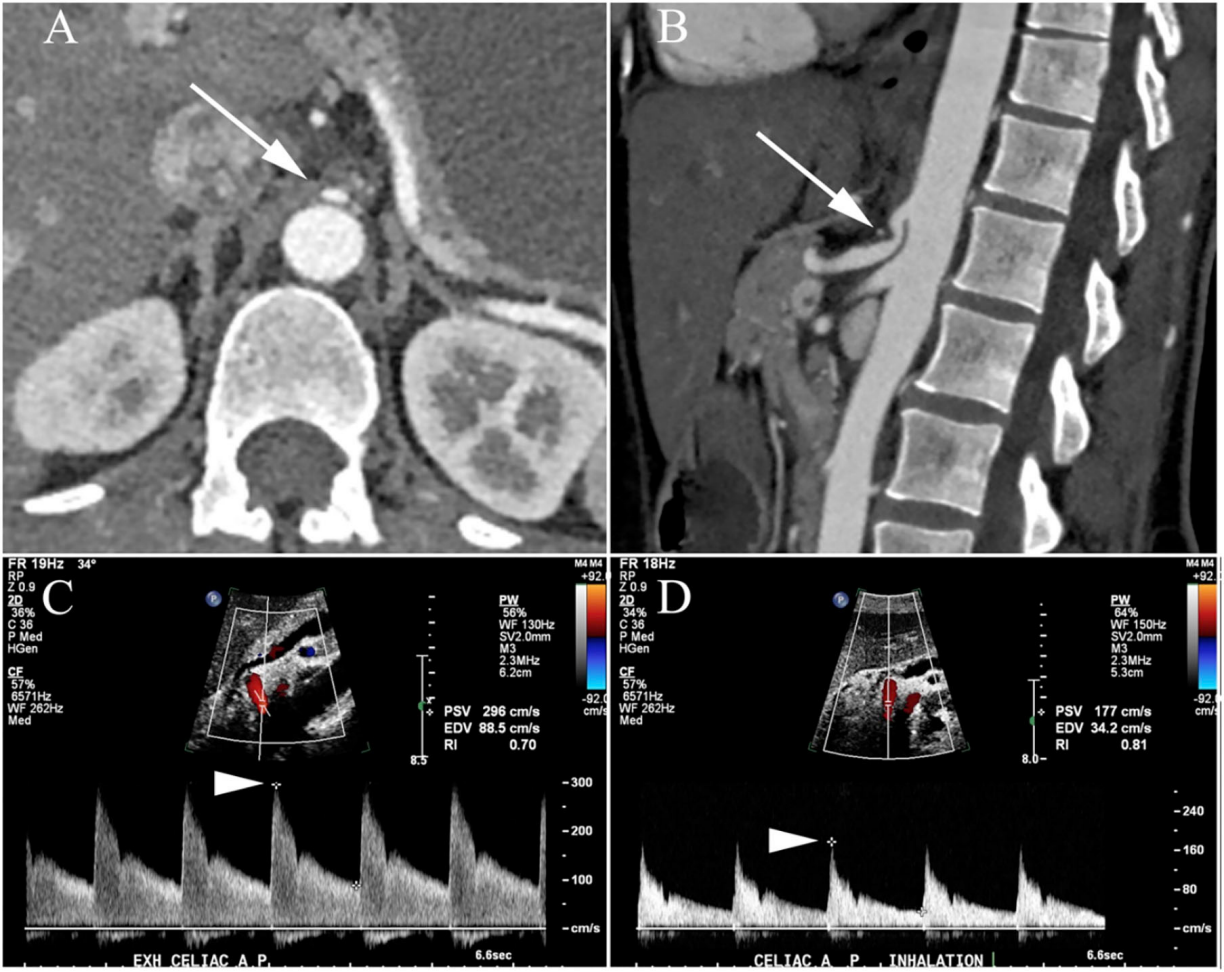
Patient with neurogenic median arcuate ligament syndrome (nMALS). Axial **(A)** and sagittal **(B)** CTA images demonstrate severe narrowing of the celiac artery origin (arrow) related to extrinsic compression by the median arcuate ligament. Duplex ultrasound demonstrated normal arterial flow during expiratory ventilation with a peak systolic velocity (PSV) of 286 cm/s (arrowhead, **C**) but marked flow impairment during inspiration with PSV of 177 cm/s (arrowhead, **D**) equivalent to >70% stenosis and hemodynamic compromise.

A 44-year-old woman with clinically diagnosed hypermobile Ehlers-Danlos syndrome (EDS), confirmed by a geneticist with expertise in connective tissue disorders and based on physical examination findings along with negative genetic testing, presented to Neurogastroenterology Clinic with complaints of nausea, oral tingling, rhinorrhea, tinnitus, and urgent diarrhea with certain foods, abdominal bloating, distention, pain, and constipation. She also reported frequent dizziness and lightheadedness with standing, though without any episodes of true syncope. She was found to have positive titers of N-type calcium channel autoantibody (0.15 nmol/L vs. <0.03 nmol/L), elevated ANA (1:160) and a lip biopsy (focal lymphocytic sialadenitis) consistent with a diagnosis of seronegative Sjögren's Syndrome. For evaluation of her abdominal pain, she underwent a series of studies to look for neurogenic median arcuate ligament syndrome (nMALS). Her duplex ultrasound revealed stenosis of the celiac artery, a CTA of her abdomen demonstrated a lower lying median arcuate ligament that was closely approximating the origin of the celiac artery during both inspiration and expiration (Figure 5). There was also evidence of collateral artery formation noted on CTA. An MRI protocolized to identify the celiac ganglia was not approved by insurance. However, she had a dramatic improvement in her abdominal pain immediately following a CT guided celiac plexus block, confirming the diagnosis of nMALS. An MRI brain revealed bifrontal white matter signal abnormalities consistent with neurologic involvement of her seronegative Sjögren's Syndrome.

Because she also reported symptoms of oral tingling, tinnitus, and urgent diarrhea, she underwent upper endoscopy with mucosal biopsy sampling. Gastrointestinal mucosal biopsy revealed elevated mast cell counts (via CD-117 staining) in the stomach (gastric antrum; 20.4 mast cells per 10 high-powered fields (HPFs); gastric body, 20 mast cells per 10 HPFs) and duodenum (proximal duodenum, 31 mast cells per 10 HPFs; distal duodenum, 21.1 mast cells per 10 HPFs). Her laboratory testing also revealed an elevated plasma histamine. These results were consistent with a diagnosis of mast cell activation syndrome (MCAS).

**Figure 6:**
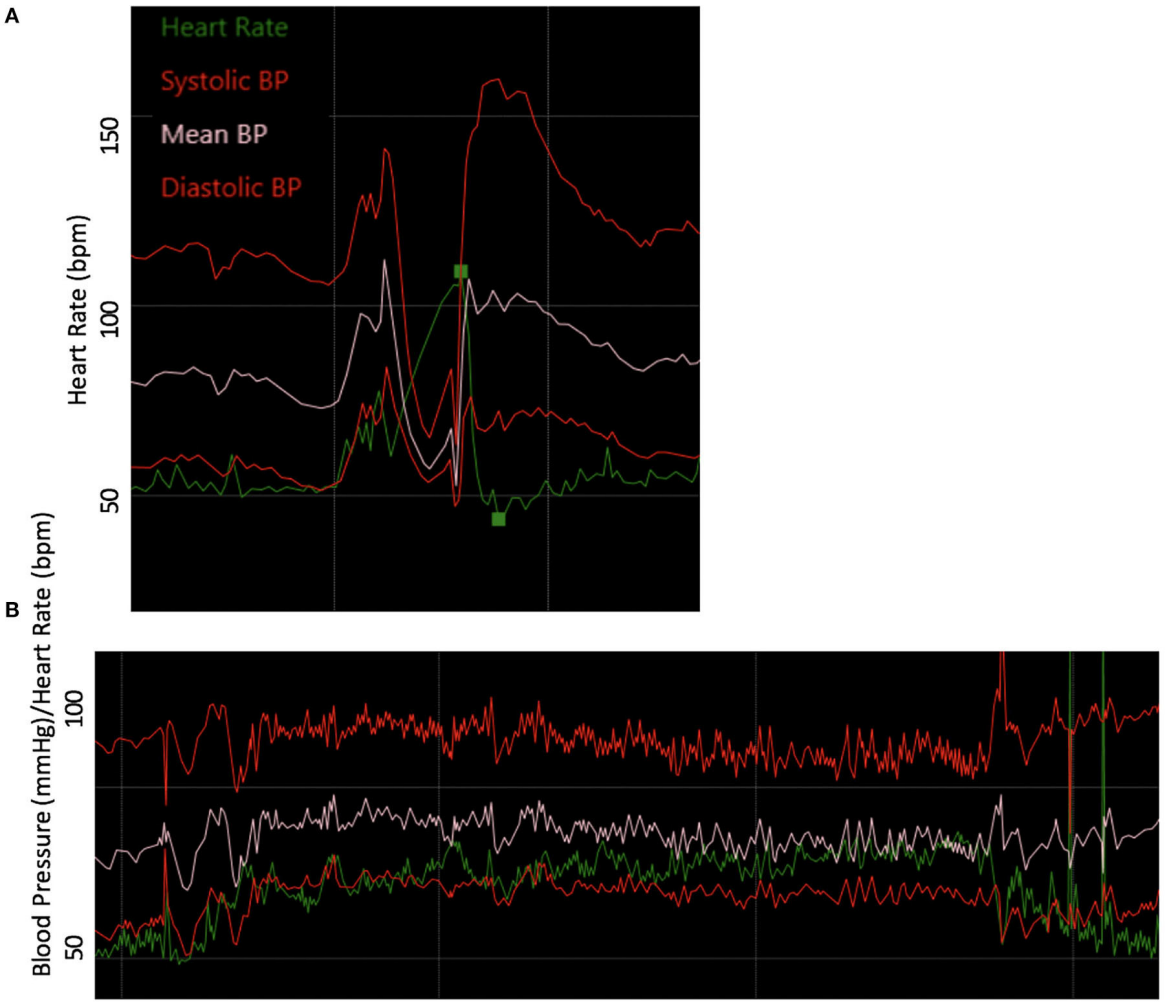
Abnormal autonomic testing reveals poor vasomotor tone. **(A)** Blood pressure responses to the Valsalva maneuver were notable for blunted late phase II. **(B)** The blood pressure and heart rate responses to 10- min head-up tilt accompanied by modest drop in blood pressure and heart rate increment.

Autonomic reflex testing, including 70° HUT, Q-SWEAT, and quantitative pupillometry, did not reveal an overt autonomic neuropathy, although it did demonstrate blunted late phase II blood pressure response to Valsalva and a COMPASS-31 score of 44.85 (domain scores: Orthostatic intolerance 7, Vasomotor 0, Secretomotor 0, Gastrointestinal 17, Bladder 0, and Pupillomotor 5). Current standardized autonomic reflex testing primarily focuses on the cardiovascular complications of autonomic nervous system disorders. Her testing did reveal blunted late phase II blood pressure response to Valsalva, which may be related to underlying seronegative Sjögren's Syndrome, MCAS, or EDS (Figure 6). To address her symptoms of orthostatic dizziness, she was started on NormaLyte oral rehydration salts with significant reduction in her orthostatic symptoms. To address her MCAS, she was started on ranitidine, Montelukast, cetirizine, ketotifen, and cromolyn sodium with dramatic improvement in MCAS related symptoms, including many of her neurologic complaints.

Given that she had near complete, but temporary, resolution of her chronic abdominal pain with a CT guided celiac plexus block she was diagnosed with nMALS and referred for surgical decompression of the celiac ganglia and plexus. Surgical decompression completely resolved her chronic abdominal pain.

Interestingly, patients with genetic hypermobile connective tissue disorders have increased co-morbid gastrointestinal symptoms, gastrointestinal motility disorders (197), neurologic disorders, immune mediated disorders, and neuroimmunologic disorders. The mechanism by which genetic connective tissue disorders lead to the development of gastrointestinal symptoms are not completely understood. Gastrointestinal dysmotility, nMALS, and MCAS are very common comorbid illnesses within this patient population and this comorbid illness burden further complicate their care.

In this case, we were able to diagnose seronegative Sjögren's syndrome and detect N-type calcium channel autoantibodies, raising concern for neurological autoimmune disease and recommended immunomodulatory therapy, which she has declined to date, given the above overall improvement in her symptom burden. Utilizing multi-modal imaging techniques and a diagnostic celiac plexus block, we were able to support a diagnosis of nMALS (Figure 5) for which she has successfully undergone surgical decompression with complete resolution of abdominal pain, bloating, distention, and constipation. MCAS is a common occurrence in EDS patients and very commonly presents with numerous gastrointestinal and systemic symptoms (198). MCAS is defined by aberrant activation of mast cells resulting in excessive secretion of mast cell mediators including histamine, leukotrienes, and prostaglandins. The pathophysiologic mechanism that leads to higher rates of MCAS within the EDS population is not yet known. Importantly, treatment with widely available, over the counter agents that suppress mast cell effector molecules, provide significant relief from symptoms in many patients.

## Conclusion

Patients with inflammatory and autoimmune neurological disorders often have comorbid gastrointestinal symptoms that are overlooked. Providers in both Neurology and Gastroenterology should no longer be satisfied with labeling patients with functional disorders, especially when these patients exhibit multi-systemic symptoms. In such cases, both neurologists and gastroenterologists may individually lack the training or capacity to address these cross-disciplinary disorders, and therefore should work in close collaboration. Case 1 demonstrated the dangers of missing a diagnosis of autoimmune limbic encephalitis by labeling the patient with a functional nausea and vomiting disorder. Case 2 highlighted the quality of care a patient can receive when clinicians take the time to integrate all the information they receive from clinical testing, as the PET-CT revealed not only myositis, but also profound gastroparesis. Furthermore, this patient met criteria for idiopathic gastroparesis, which he clearly did not have as his gastrointestinal dysmotility great improved with therapies targeted to treat his myositis. Case 3 highlighted the underappreciated neuroimmunological and gastrointestinal co-morbidities of patients with connective tissue disorders, and the utility of surgical and mast cell-based interventions. Importantly, we show examples of patients with immune mediated neurogastrointestinal disorders who were responsive to a combination of symptomatic management and immune-modulating therapy, which markedly alleviated symptoms and improved their quality of life. Finally, following expanded diagnostic investigation, therapies targeted at the underlying pathophysiology of the disease process may have the ability to prevent further disease progression along with reducing systemic complications.

## Challenges and Future Directions

Neuroimmune etiologies for gastrointestinal dysfunction are underappreciated and patients with such conditions are often misdiagnosed as having functional disorders. Unfortunately, these patients may be subject to a diagnostic odyssey before identifying their underlying condition. The extensive workup required to diagnose these patients is a significant clinical challenge (Table 2). In the setting of AGID, specific autoantibodies (listed in Table 1) are highly associated with the disease and should increase clinical suspicion of this entity. However, in other neuroimmune-mediated conditions affecting the gastrointestinal tract, serological markers may be absent or may be not be clearly defined. The above cases highlight the importance of broad testing using a variety of modalities to evidence neuroinflammatory processes, though further investigation is required to define more specific diagnostic markers.

Given the lack of clinical detection of neuroimmune-mediated gastrointestinal dysfunction, investigation into treatment of such conditions has been limited to case reports and retrospective studies. The use of immunomodulatory agents, including IVIG and corticosteroids, carry risks of opportunistic infections among other side effects. Thus, such treatment should be reserved for patients that exhibit significant gastrointestinal symptoms that are not appropriately managed with symptomatic treatments. Randomized, placebo-controlled, double-blind clinical trials are needed for the variety of neuroinflammatory gastrointestinal dysmotility conditions.

## Ethics Statement

We would like to confirm that written informed consent was obtained from the individual(s) AND/OR minor(s)' legal guardian/next of kin for the publication of any potentially identifiable images or data included in this article.

## Author Contributions

JS was responsible for literature review and manuscript preparation. JM was responsible for interpretation of data and critical revision of manuscript for intellectual content. MC was responsible for study concept and design, interpretation of data, and critical revision of manuscript for intellectual content. JH was responsible for critical revision of manuscript for intellectual content. LP and SC were responsible for study concept and design, interpretation of data, literature review, and critical revision of manuscript for intellectual content. All authors contributed to the article and approved the submitted version.

## Conflict of Interest

The authors declare that the research was conducted in the absence of any commercial or financial relationships that could be construed as a potential conflict of interest.
